# Follicular development of canine ovaries stimulated by a combination treatment of eCG and hCG

**DOI:** 10.1002/vms3.121

**Published:** 2018-10-01

**Authors:** Maki Hirata, Fuminori Tanihara, Masayasu Taniguchi, Mitsuhiro Takagi, Tsukasa Terazono, Takeshige Otoi

**Affiliations:** ^1^ Faculty of Bioscience and Bioindustry Tokushima University Myozai‐gun Tokushima Japan; ^2^ Joint Faculty of Veterinary Science Yamaguchi University Yamaguchi‐shi Yamaguchi Japan

**Keywords:** dog, oestrus induction, PMSG, transplantation, ultrasonography

## Abstract

Ovarian follicular dynamics is not well known in dogs. Imaging of ovaries is technically difficult; however, ovaries clamped at a subcutaneous site can more easily be monitored using ultrasound imaging. This study investigated the follicular development of canine ovaries stimulated by hormone treatment using ultrasound imaging of the ovaries clamped at a subcutaneous site. Oestrus was induced using subcutaneous administration of 500 IU equine chorionic gonadotropin (eCG) and 1000 IU human chorionic gonadotropin (hCG) (eCG/hCG). Five bitches were given 1000 IU hCG 11 days after eCG/hCG administration. Examinations with ovarian ultrasonography using a 7.5‐MHz sector transducer, vaginal cytology, and assays of serum oestrogen and progesterone were performed daily until 20 days after eCG/hCG administration. Serosanguineous vaginal discharges and vaginal cytology of two of the bitches were observed. New follicular growth (>1.0 mm in diameter) was observed in all bitches from 2 to 8 days after eCG/hCG administration. The mean diameter of follicles and maximum numbers of follicles per ovary ranged from 2.8 to 5.5 mm and 4 to 16, respectively. The elevation in oestrogen concentrations after eCG/hCG administration was observed in all bitches, and elevation in progesterone concentration (>2 ng mL^−1^) was observed in three bitches. However, no follicles ovulated until 9 days after hCG administration. In conclusion, although the number of examined bitches were limited, follicular growth in ovaries clamped at a subcutaneous site can be monitored using ultrasound imaging. Ovarian ultrasonography showed that eCG/hCG administration induced new follicular growth and hCG administration induced increases in oestrogen concentrations but not ovulation by hCG administration.

## Introduction

For many mammalian species, including the dog, knowledge of ovarian follicular dynamics, for example the interval required for follicle growth and the hormonal responsiveness of follicles, is not well known. The development of a method to induce oestrus effectively and predictably in bitches would be advantageous in the clinical management of prolonged anoestrus or infertility. To date, it has been demonstrated that oestrus of bitches can be induced by the administration of a variety of hormones, including porcine FSH (Bouchard *et al*. 1991), equine chorionic gonadotropin (Arnold *et al*. 1989; Kusuma & Tainturier 1993) and GnRH analogues (Concannon 1989; Inaba *et al*. 1998). Of these, equine chorionic gonadotropin (eCG or PMSG) has been most frequently used as a gonadotropin for the induction of oestrus because of the ready availability of eCG in most countries. The use of eCG has been more successful for oestrus induction in bitches than the use of FSH. In addition, the combination of eCG and human chorionic gonadotropin (hCG) has been used to induce oestrus in bitches (Stornelli *et al*. 2012). However, it has been suggested that the most frequent problems encountered with eCG use arise from the unpredictability of an individual bitch's response, either in the number of follicles that develop or in the percentage of bitches that initiate follicular growth (Kutzler 2007).

The reproductive biology of the bitch is known to be unique. For instance, ovulation occurs approximately 2–3 days after the luteinizing hormone (LH) surge and, prior to ovulation, the serum progesterone concentration begins to increase from basal values coincident with the LH peak (Wildt *et al*. 1979). There is information available about the time of ovulation, but there is limited information about follicular growth through anoestrus and pro‐oestrus. Moreover, follicular growth in bitches has been usually detected indirectly through behavioural observations, vaginal smears and blood hormonal assays. Although real‐time ultrasonography can reveal the development of canine ovarian follicles (Boyd *et al*. 1993; Hase *et al*. 2000), no method has been established to determine or predict ovulation accurately. The location and the small size of ovaries make imaging technically difficult, restricting progress in our understanding of follicular development in bitches. Difficulty in imaging the ovaries especially in anoestrus has been noted (England & Allen 1989; Hase *et al*. 2000). However, when ovaries are clamped at a subcutaneous site, their follicular growth can more easily be monitored using ultrasound imaging.

The objectives of the present study were to evaluate the follicular development, hormonal profiles and oestrous behaviour in bitches treated with a combination of eCG and hCG, in which the ovaries were clamped at a subcutaneous site for ultrasound imaging of follicular growth.

## Materials and methods

### Animals

Beagle bitches (aged 5–7 years; mean weight, 9.0 ± 1.5 kg) in a closed breeding colony were used for this study. The dogs were housed individually in stainless steel cages (900 × 770 × 710 mm) and were given standard commercial dog food once a day and water *ad libitum*.

### Ovary clamp at subcutaneous site

Bilateral malacotomy of five bitches was performed using a ventral‐flank abdominal approach with routine techniques and materials. General anaesthesia was induced with an intravenous injection of 0.2 mg kg^−1^ midazolam hydrochloride (Astellas Pharma Inc., Tokyo, Japan) mixed with 0.2 mg kg^−1^ butorphanol tartrate (Meiji Seika Co. Ltd., Tokyo, Japan), followed by 4 mg kg^−1^ propofol (Fuji Pharmaceutical Co. Ltd., Toyama, Japan). After endotracheal intubation, the dog was mechanically ventilated with isoflurane in pure oxygen. The uterine artery and vein were then ligated and severed at the cranial tip of the uterine horn, after which the ovary was removed from the uterus. After removal of the ovary, each ovary that maintained blood circulation from the suspensory ligament was clamped at a subcutaneous site through the external abdominal oblique muscle. Briefly, the external abdominal oblique muscle was incised and a grid approach (blunt dissection) was used to go through the internal oblique and transverse muscles. Then, the peritoneum was incised carefully to give access to the peritoneal cavity. The ovary was gently exteriorized on the respective side and placed superficially to the external abdominal oblique muscle, taking care not to strangulate the ovarian blood supply. Finally, the subcutaneous layer and skin incision were closed.

### Hormonal treatment and ovarian ultrasonography

To induce oestrus, the bitches were subcutaneously administered 500 IU eCG (Kyoritu Seiyaku, Tokyo, Japan) and 1000 IU hCG (Kyoritu Seiyaku) (eCG/hCG). Each bitch was given a further 1000 IU hCG at 11 days after eCG/hCG administration.

All dogs were examined every day for the presence of vulval swelling, and serosanguineous vaginal discharge. Vaginal smears and follicular development in each bitch were monitored once daily from 1 day before eCG/hCG administration until Day 20 (Day 1 = eCG/hCG injection). Vaginal smears were stained using Giemsa (Merck, Armstadt, Germany) and were evaluated for cell types and approximate percentage of epithelial cells, as previously described (Concannon *et al*. 2001). The largest diameter of each follicle was measured by the Prosound *α*7 ultrasound scanner (ALOKA Co., Ltd., Tokyo, Japan) equipped with a 6.0–13.0 MHz linear‐array transducer.

### Hormonal assay and oestrous cycle

Blood samples were collected from the jugular vein every day, starting from 1 day before eCG/hCG administration until Day 20; the samples were collected in vacutainers and centrifuged at 1500*g* for 15 min. The serum was separated and stored at −30°C until the sample was assayed for oestrogen and progesterone. The concentrations of serum oestrogen and progesterone were measured by commercially available quantitative sandwich enzyme‐linked, immunosorbent assay (ELISA) kits (DRG Estradiol ELISA, EIA 2693, and DRG Progesterone ELISA, EIA 1561, NJ, USA). The intra‐assay and interassay coefficients of variation were below 6.81% and 9.39% for the oestradiol ELISA, respectively, and the sensitivity was 9.71 pg mL^−1^. The intra‐assay and interassay coefficients of variation were below 6.99% and 9.96% for the progesterone ELISA, respectively, and the sensitivity was 0.045 ng mL^−1^.

At the commencement of this study, the oestrous cycle of each dog was determined, based on serum concentrations of progesterone and oestradiol and the vaginal superficial cell index: pro‐oestrus (>50% superficial cells by cytology and basal or rising serum concentrations of progesterone and oestradiol), dioestrus (<30% superficial cells and serum concentrations of >1.0 ng mL^−1^ progesterone and <30 pg mL^−1^ oestradiol) or anoestrus (<30% superficial cells and serum concentrations of <1.0 ng mL^−1^ progesterone and <10 pg mL^−1^ oestradiol (Concannon *et al*. 2001; Concannon 2011).

## Results

**Figure 1:**
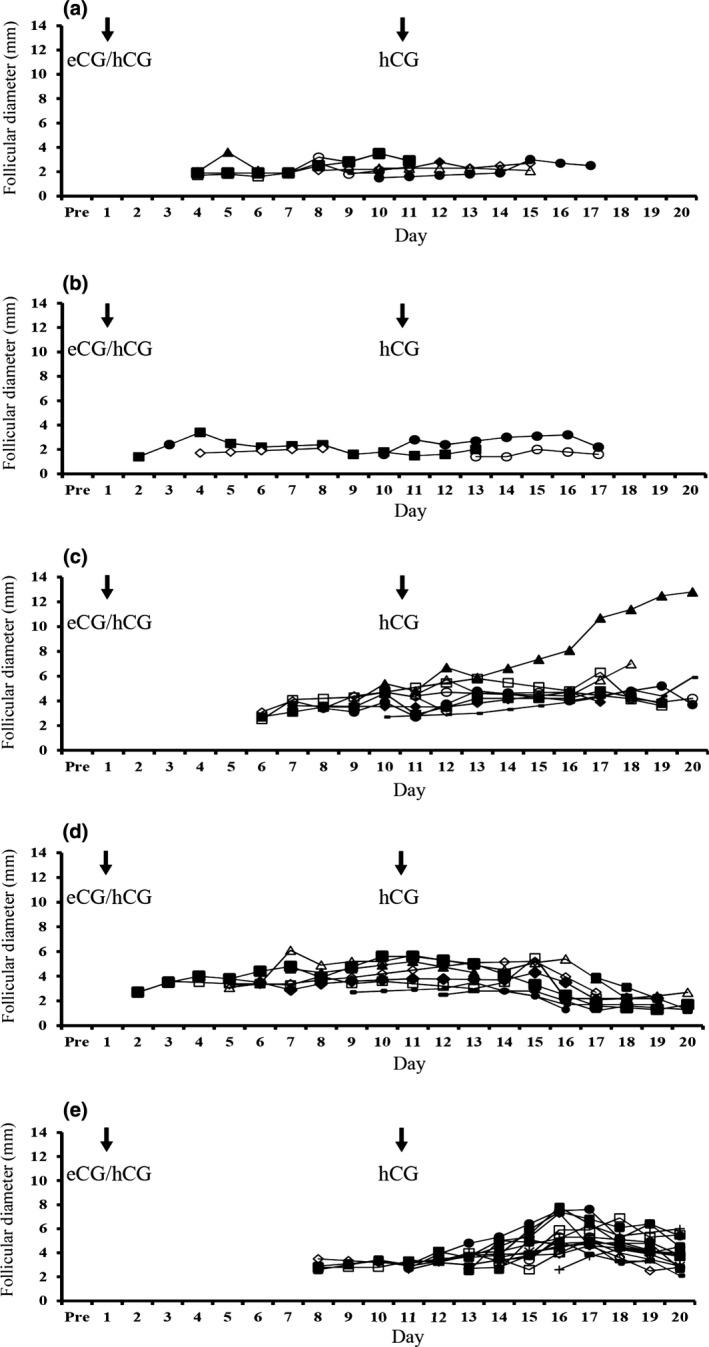
Profiles of total visible follicles (largest diameter >1 mm) in ovaries after eCG/hCG administration. Arrows indicate the time of eCG/hCG and hCG administration.

**Figure 2:**
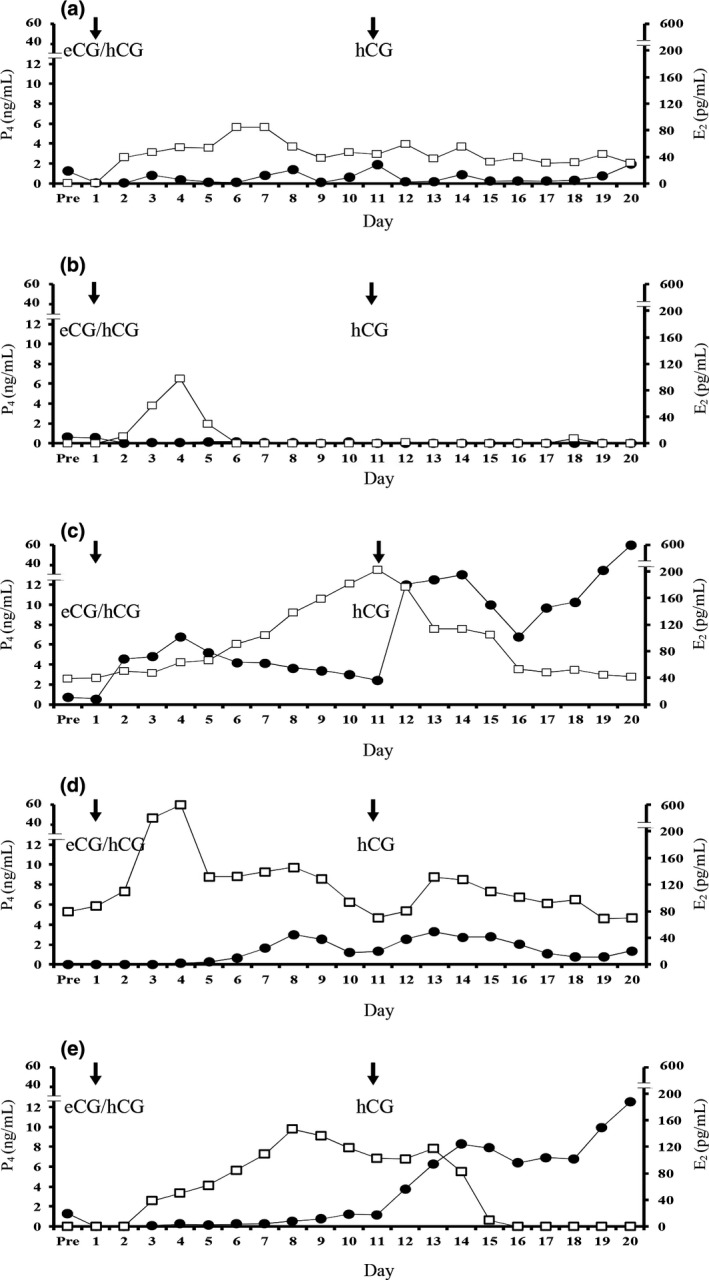
Profiles of serum oestrogen (‐□‐) and progesterone (‐●‐) after eCG/hCG administration. Arrows indicate the time of eCG/hCG and hCG administration.

Five bitches at the anoestrous (A, B and E) and pro‐oestrous (C and D) stages of the oestrous cycle were administered eCG/hCG. Of these, two bitches (A and D) showed vulval oedema and serosanguineous discharges at 6 days after eCG/hCG administration, and another two bitches (C and E) showed only vulval swelling at 4 days and 7 days, respectively, but one bitch (B) did not show any signs of oestrus. Follicular growth (>1.0 mm in diameter) was observed in all bitches after eCG/hCG administration, and new follicular growth appeared from 2 to 8 days after eCG/hCG administration (Fig. 1). The mean diameter of follicles and maximum numbers of follicles ranged from 2.8 to 5.5 mm and 4–16 per ovary, respectively. However, no follicles ovulated until 9 days after the second hCG administration. The elevation in oestrogen concentrations after eCG/hCG administration was observed in all bitches, and the elevation in progesterone concentrations (>2 ng mL^−1^) was observed in three bitches (C, D and E) (Fig. 2).

## Discussion

A better understanding of folliculogenesis in the bitch is required for the development of reproduction biotechnologies and clinical management. However, folliculogenesis in bitches has been far less documented than that of cows, ewes or that of laboratory animals (mice, rats). In this study, bitches at the anoestrous (three heads) and pro‐oestrous (two heads) stages of the oestrous cycle were used for the induction of oestrus. The follicular development in bitches after eCG/hCG administration was followed by ultrasound imaging the ovaries clamped at a subcutaneous site. It has been suggested that the stage of the oestrous cycle affects response to hormonal treatment including eCG (Renton *et al*. 1981). Actually, we observed a variety of physiological and endocrinological changes after hormonal treatment in each bitch, but follicular growth was observed in all bitches and new follicular growth appeared from 2 to 8 days after the eCG/hCG administration.

The administration of eCG has been used with protocols ranging from daily to weekly injections using either subcutaneous or intramuscular routes. It has been reported that individual variations in the number of follicles which develop after the eCG protocol are unpredictable (Kutzler 2007). Moreover, premature luteal failure with subsequent shortening of dioestrus and pregnancy loss has been reported from the use of eCG (Nakao *et al*. 1985). It has been demonstrated that the combination of hCG with eCG at the start of treatment for the induction of oestrus had a better ovulatory response than treatment with eCG alone (Wright 1980). In the present study, therefore, we used the combination of hCG and eCG to induce oestrus in these bitches. All bitches showed increases in oestrogen concentrations after eCG/hCG administration and increases in progesterone concentrations were observed in three bitches. However, we could not observe ovulation in all bitches, and all follicles regressed irrespective of hCG administration. When we continued the observation of follicular growth every 10 days from 20 to 60 days (post eCG/hCG injection), the formation of the corpus luteum could not be observed in all animals (data not shown). It has been suggested that the induced oestrous periods differ from spontaneous cycles, and serum oestrogen concentrations are generally greater and progestogen concentrations lower in induced cycles (England & Allen 1991). The use of eCG with high doses for oestrus induction has been shown to produce an endogenous hyperoestrogenism that is associated with implantation failure (Lehmann *et al*. 1975; Arnold *et al*. 1989). Barta *et al*. (1982) also reported that treatment with eCG induces a progressive decline in progesterone concentrations after oestrus. On the other hand, Stornelli *et al*. (2016) suggested that a single injection of 50 IU kg^−1^ of eCG in late anoestrous bitches successfully induced changes in follicular growth. Moreover, a treatment with 50 IU kg^−1^ of eCG combined 7 days later with 500 IU of hCG can induce normal and fertile oestrus in bitches (Stornelli *et al*. 2012). In this study, we used similar concentration of eCG for oestrus induction but administered hCG at time of eCG treatment in anticipation of a synergistic effect for follicular development. Moreover, we administered a higher concentration (1000 IU) of hCG at 11 days after eCG/hCG administration for induction of ovulation. Nickson *et al*. (1992) reported that superovulation could be induced by the combination of eCG and hCG, but the ovulation rate was poor. For gonadotropin‐induced cycles, there were large numbers of small follicles produced, but many of the follicles did not ovulate (England *et al*. 2009). To date, many protocols for oestrus induction in bitches include hCG use, but the administration of an ovulation induction agent as part of an oestrus induction protocol has been considered to be controversial. Treatment with hCG on the first and third days of oestrus has been reported to prolong behavioural oestrus and suppress progesterone secretion (Cirit *et al*. 2007). In the present study, the reason for the lack of an ovulatory response to hCG administration remains unclear, but the response of these ovaries might result from administration with overdose of hCG‐ or gonadotropin‐induced cycles.

In conclusion, the observation of follicular growth by ultrasonography showed that eCG/hCG administration induced new follicular growth and increases in oestrogen concentrations but not subsequent ovulation by hCG administration. Further studies are needed to clarify follicular growth and to determine the optimal treatment for induction of ovulation.

## Source of Funding

This work was supported in part by the Ministry of Education, Culture, Sports, Science and Technology (No.17H03938).

## Conflict of Interest

The authors declare that there is no conflict of interest.

## Ethics statement

The authors confirm that the ethical policies of the journal, as noted on the journal's author guidelines page, have been adhered to and the appropriate ethical review committee approval has been received. All the animals involved in this study received humane care in compliance with the Guide for the Care and Use of Laboratory Animals prepared by the Institute of Laboratory Animal Resources, National Research Council. All procedures were approved by the Animal Research Committee of Yamaguchi University.

## Contribution

M.H., F.T. and T.O. conceived the study and wrote the manuscript. M.H., F.T. and T.T. performed the majority of experiments. F.T. designed the study, coordinated all of the experiments, and reviewed the manuscript. T.T. performed the ovary clamp and ovarian ultrasonography. M.T. and M.T. performed hormonal assay and revised the manuscript. All of the authors read and accepted the manuscript.
